# Feasibility and basic acoustic characteristics of intelligent long-term bowel sound analysis in term neonates

**DOI:** 10.3389/fped.2022.1000395

**Published:** 2022-11-03

**Authors:** Ping Zhou, Meiling Lu, Ping Chen, Danlei Wang, Zhenchao Jin, Lian Zhang

**Affiliations:** ^1^ Department of Neonatology, Baoan Women's and Children's Hospital, Shenzhen, China; ^2^ Research and Development Department, Linkwah Integrated Circuit Institute, Nanjing, China

**Keywords:** neonate, bowel sounds, convolutional neural networks, artificial intelligence, acoustic parameter

## Abstract

**Objective:**

Bowel dysfunction continues to be a serious issue in neonates. Traditional auscultation of bowel sounds as a diagnostic tool in neonatal gastrointestinal problems is limited by skill and inability to document and reassess. Consequently, in order to objectively and noninvasively examine the viability of continuous assessment of bowel sounds, we utilized an acoustic recording and analysis system to capture bowel sounds and extract acoustic features in term neonates.

**Methods:**

From May 1, 2020 to September 30, 2020, 82 neonates who were hospitalized because of hyperbilirubinemia were included. For 20 h, a convolutional neural network-based acoustic recorder that offers real-time, wireless, continuous auscultation was employed to track the bowel sounds of these neonates.

**Results:**

(1) Usable data on five acoustic parameters of bowel sound was recorded for 68 neonates, and the median values were as follows: The rate was 25.80 times/min [interquartile range (IQR): 15.63–36.20]; the duration was 8.00 s/min (IQR: 4.2–13.20); the amplitude was 0.46 (IQR: 0.27–0.68); the frequency was 944.05 Hz (IQR: 848.78–1,034.90); and the interval time was 2.12 s (IQR: 1.3–3.5). (2) In comparison to the parameters of the bowel sounds recorded from the right lower abdomen in 68 infants, the acoustic parameters of the 10 out of 68 infants from chest controls and blank controls were considerably different. (3) The 50%–75% breast milk intake group had the highest rate, the longest duration, and the highest amplitude of bowel sounds, while the >75% breast milk intake group had the highest frequency of bowel sounds. (4) Compared with neonates without hyperbilirubinemia, there was no significant difference in the five parameters of bowel sounds in hyperbilirubinemia infants; nor was there a significant effect of phototherapy and non-phototherapy status on the parameters of bowel sounds during bowel sound monitoring in hyperbilirubinemia patients. (5) A mild transient skin rash appeared on the skin of three infants. No other adverse events occurred.

**Conclusion:**

The acoustic recording and analysis system appears useful for monitoring bowel sounds using a continuous, invasive, and real-time approach. Neonatal bowel sounds are affected by various feeding types rather than hyperbilirubinemia and phototherapy. Potential influencing factors and the significance of their application in neonatal intestinal-related disorders require further research.

## Introduction

Bowel sounds are regarded as an essential signal to monitor gastrointestinal function since they are an objective representation of the status of gastrointestinal motility. Due to the features of weak signal, strong noise, individual variability, and strong randomness, it is challenging to measure and run long-term monitoring in clinical practice (1, 2). In terms of sound recording and visualization, modern auscultation employing digital stethoscopes is superior to traditional approaches. For prolonged auscultation, modern digital stethoscopes are too heavy and nonconformal to the skin. Inaccurate diagnoses result from friction noise and motion artifacts from the stiffness (3). The new continuous acoustic recorder employs Bluetooth transmission technology to gather data and a deep neural network algorithm model to achieve better noise reduction, lower power consumption, higher recognition rate, and continuous monitoring (4, 5). It can provide the sound waveform and spectrum of bowel sounds to accurately and impartially reflect intestinal motility (6). The approach has been used in adult medicine and surgery to monitor the recovery of bowel function after surgery, diagnose acute peritonitis, identify early intestinal obstruction, and diagnose diarrheal disease (7, 8).

Due to neonatal immaturity and developing gastrointestinal systems, preterm infants are more prone to feeding intolerance and necrotizing enterocolitis (NEC). Near-infrared spectroscopy (9) and intestine ultrasonography (10) are two non-invasive methods that have been examined for blood flow and motility to observe intestinal movement and aid in the diagnosis of NEC in preterm infants. However, the necessity for expert interpretation and the incapacity of real-time and long-term monitoring restrict their extensive application. Although it has been reported (11), electronic stethoscopes cannot be used for long-term monitoring due to inconvenient use and a limited number of parameters.

The convolutional neural network (CNN) and artificial intelligence-based acoustic recording and analysis systems have shown good promise in adults, but there are no reports of their application in neonates. In order to do a preliminary validation of the use feasibility in neonates and to gather certain fundamental parameters of bowel sounds, we created this investigative pilot study.

## Materials and methods

### Study population

The study's participants were chosen from among neonates born between May 1 and September 30, 2020, who had hyperbilirubinemia. The following criteria are required for inclusion: (1) term neonates; (2) hospitalization for mild or moderate hyperbilirubinemia; and (3) stable vital signs. Premature infants, severe hyperbilirubinemia, and complications from diseases that may affect gastrointestinal function, such as congenital heart disease, gastrointestinal tract malformations, enteritis, and systemic infection, are all excluded. Healthy infants would be ideal study subjects, but in practice, it was not possible to conduct long-term monitoring because they are routinely discharged from hospital 24 to 48 h after birth. While being tested, participants fed normally and their vital signs remained stable. Despite the fact that neither hyperbilirubinemia nor phototherapy has been shown to significantly alter gastrointestinal motility in neonates (12), a negative impact is still possible. Hence, in the data analysis phase, we added 10 term infants without hyperbilirubinemia to do a comparative analysis. The following data was gathered by one doctor, and it was verified by another: gestational age, birth weight, gender, age, breast milk amount, and total bilirubin. This single-center, observational study was conducted in the Department of Neonatology at Shenzhen Baoan Women's and Children's Hospital and was approved by the hospital ethics committee (Approval No. LLSCHY 2020-07-09).

### Principles of long-term bowel sound monitoring

As previously reported by the author Yin et al. (13), bowel sounds were captured using a high-sensitivity micro-electro-mechanical system (MEMS) sensor, and noise was significantly decreased by the sensor's micro-controller unit (MCU). Through the use of Bluetooth, data is sent to the receiver, which then uploads it to the server. Supporting software was running in the background on the server to save the data and record the necessary information. A convolutional recurrent neural network (CRNN), which is composed of a five-layer CNN network, a bi-directional gated recurrent unit network, and a fully connected layer, was constructed using the Mel Frequency Cepstrum Coefficient (MFCC) features that were extracted from the framed bowel sound data. The MFCC and the CRNN network were able to recognize specific features in the data to obtain bowel sound segments.

### The method for monitoring bowel sounds

**Figure 1:**
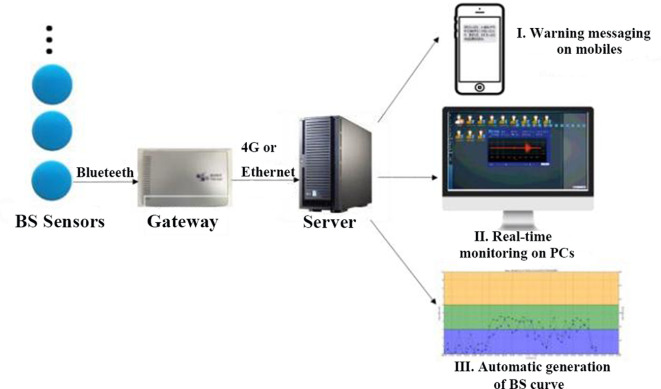
Working diagram of the bowel sound recording and analysis system.

A continuous acoustic recording and analysis system with a National Class II Medical Device Registration Certificate was used to record bowel sounds. The entire system, as depicted in Figure 1, was made up of three components, as previously used (14): (1) wireless sensors to collect bowel sounds in real time; (2) gateways to transmit the data from multiple bowel sound sensors to remote servers; and (3) servers to analyze the bowel sound data and distribute the processing result on terminals. Six bowel sound sensors were able to be connected *via* a single gateway using the Bluetooth protocol. Gateways sent information about bowel sounds by 4G.

**Figure 2:**
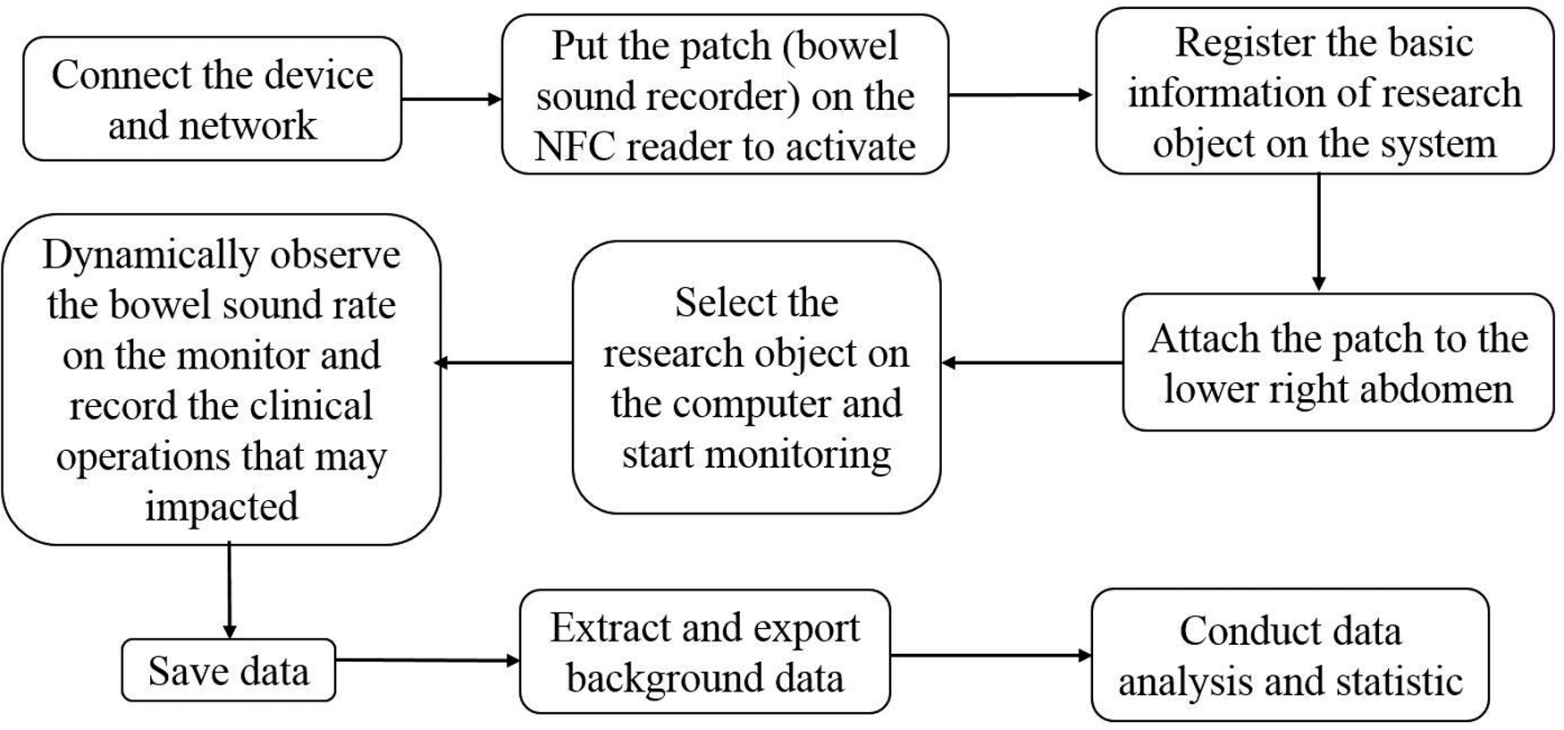
The operational flow chart for collecting and analyzing bowel sounds.

The practical procedure is described as follows in Figure 2.

**Figure 3:**
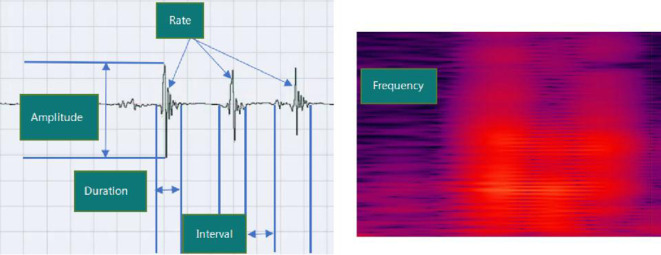
Schematic diagram and the definitions of five acoustic parameters of bowel sounds. Rate (times per minute): the number of bowel sounds recorded per minute. Duration (s/min): the time it takes for bowel sounds to last one minute. Amplitude: the loudness of sound (in the range of 0–1). Frequency (Hz): acoustic frequency that is dominant; Interval (s): the average time of between sounds interval in a minute.

**Figure 4:**
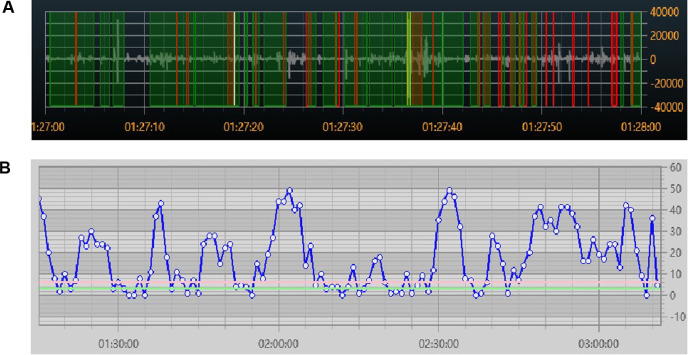
Real-time visual display of bowel sound rate (**A**) colored by bowel sounds (red), speech (green), and friction or other noises (yellow); (**B**) point-curve by minutes.

The continuous acoustic recorder sampled signals for 20 h at 4 kHz with 16-bit quantization. Five bowel sound characteristics can be collected, and Figure 3 shows their schematic diagrams and definitions. One of these, the bowel sound rate, was displayed on the screen in various colors (Figure 4A). In the same time frame, it was also explicitly represented as a point-curve (Figure 4B); the other four characteristics were acquired by the extraction and analysis of background data.

The adhesive sensor patch is a disposable, and its outer shell is comprised of medical-grade silicone and polycarbonate (PC), making it suitable for use in clinical settings and less prone to causing skin allergies. When applying and removing the patch from the newborn's sensitive skin, gentle technique is necessary. The patch was taken off if the patient's skin reacted allergically.

### Chest control and blank control

**Figure 5:**
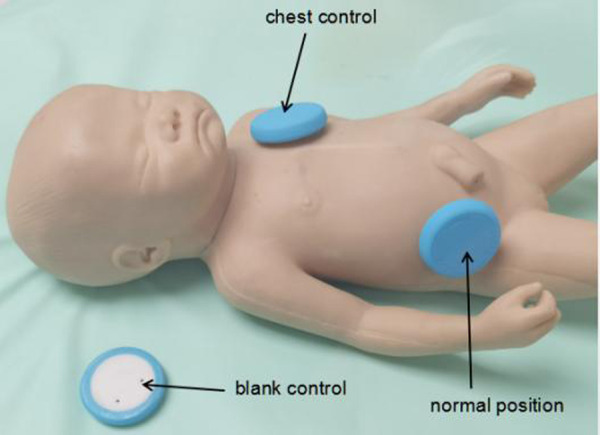
The Normal site and two control sites of the sensor.

The right lower quadrant was utilized as the typical site for bowel sound collection in this investigation because it is the most commonly used auscultation site in clinics (15). As illustrated in Figure 5, both a chest control site (sensor patch attached to the left chest) and a blank control site (sensor patch placed in the space of the incubator) were set in 10 cases to test the specificity of the acoustic recorder in collecting bowel sounds in the presence of heartbeats and ambient noise.

### Statistical analysis

Statistical analysis was performed using SPSS version 25.0 (SPSS Inc., Chicago, Illinois, United States). The mean and standard deviation were used to describe measurement data with a normal distribution, and statistical tests such as the *t*-test or analysis of variance were used to determine their significance. The median and quartile were used to describe measurement data with an abnormal distribution, and non-parametric tests were used to determine their significance. Count data was expressed by frequency and percentage and tested with the chi-square test. To obtain the fitted curve, the regression line was fitted to the scatter plots of the five bowel sound characteristics using the locally weighted regression smooth curve (Loess). Paired t-tests were used to determine the significance of bowel sound parameters in hyperbilirubinemia infants with phototherapy or non-phototherapy status. *P *< 0.05 indicated a significant difference in all statistical tests.

## Results

### Demographics

In total, 82 neonates were involved in the study. 68 neonates had 20 h of monitoring completed and produced usable data after excluding cases where the sensor fell off (*n* = 6), the sensor battery ran out (*n* = 4), or the neonates were removed from the study too soon (*n* = 4). The list of clinical features is in Table 1.

**Table 1:** Clinical features.

Characteristic	*N* = 68 patients (%)
Gestational age, mean ± SD	38.88 ± 1.24 weeks
Age, mean ± SD	4.51 ± 5.34 days
Birth weight, mean ± SD	3285 ± 372 g
Female	34 (50)
Vaginal delivery	40 (58.8)
Multiple birth	2 (2.9)
**Percentage of own mother's milk**	
<50%	40 (58.8)
50%–75%	12 (17.6)
>75%	16 (23.5)

### Basic acoustic characteristics with five parameters of bowel sounds in term neonates

**Figure 6:**
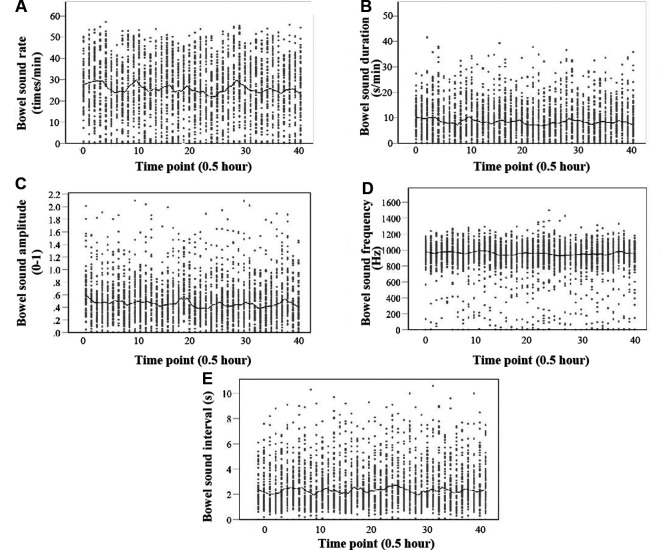
Scatter plot of five parameters of bowel sound with a locally weighted regression smoother (loess). The abscissa of the scatter plot is a time point, and every time the number increases by 1, the time increases by 30 min. (**A**) Rate (times per minute); (**B**) Duration (seconds per minute); and (**C**) Amplitude (0–1). (d) Frequency (Hz). (**E**) Interval (s).

The median values of the five bowel sound parameters among the 68 neonates with completed data were as follows, rate: 25.80 times/min (IQR: 15.63, 36.20); duration: 8.00 s/min (IQR: 4.2, 13.20); amplitude: 0.46 (IQR: 0.27, 0.68); frequency: 944.05 Hz (IQR: 848.78, 1,034.90); and interval time: 2.12 s (IQR: 1.3, 3.5). Figure 6 presents comprehensive findings.

### Comparison of bowel sounds between the normal site and two control sites

**Figure 7:**
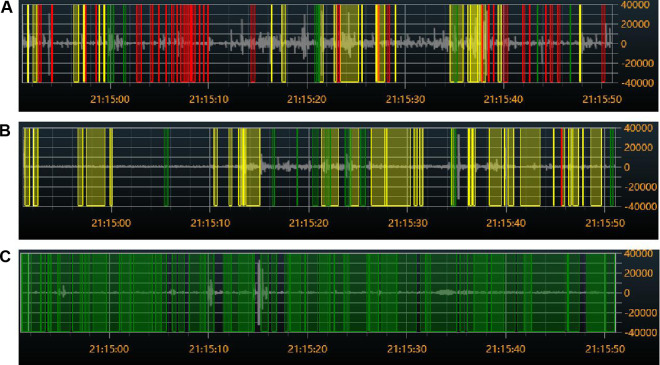
Colored visual display of bowel sound rates at different sites: (**A**) audio from the right lower abdomen. (**B**) Audio on the left side of the chest. (**C**) Audio from the blank. Red indicates bowel sounds, green indicates speech, and yellow indicates friction or other noises.

The findings from 10 cases of the chest control and blank control sites showed that there were substantial differences in each of the five bowel sound parameters when compared to the normal site in the lower right abdomen (*P* < 0.001) (Figure 7, Table 2).

**Table 2:** Comparison of bowel sounds between normal and control sites (left chest and blank).

Recording sites	Rate (times/min) *n* = 10	Duration (s/min) *n* = 10	Amplitude (0–1) *n* = 10	Frequency (Hz) *n* = 10	Interval (s) *n* = 10
Lower right abdomen	24.45 (12.60, 34.6)	7.25 (3.026, 11.88)	0.51 (0.26, 0.67)	1,000.90 (821.58, 1,076.78)	1.80 (1.00, 3.20)
Chest control	0.80 (0.30, 2.70)	0.20 (0.10, 0.60)	0.07 (0.02, 0.30)	415.15 (200.33, 727.73)	2.10 (0.50, 4.78)
Blank control	0.00 (0.00, 0.00)	0.00 (0.00, 0.00)	0.00 (0.00, 0.00)	0.00 (0.00, 4.30)	0.00 (0.00, 0.00)
*P*	<0.001	<0.001	<0.001	<0.001	<0.001

### Effect of breast milk amount on bowel sound parameters

In the comparison between groups with different percentages of breast milk amount (Table 3), the 50%–75% breast milk intake group had the highest rate, the longest duration, and the highest amplitude of bowel sounds, while the >75% breast milk intake group had the highest frequency of bowel sounds.

**Table 3:** Compares bowel sounds for various breast milk intake ratios.

Groups	*n*	Rate (times/min)	Duration (s/min)	Amplitude (0–1)	Frequency (Hz)	Interval (s)
Breast milk intake ratio	<50%	40	25.60* (13.70–35.90)	7.90* (3.60–13.20)	0.47^#^ (0.26–0.73)	941.30^#^ (837.13–1,027.98)	2.10^#^ (1.30–3.40)
	50%–75%	12	29.10^+^ (18.83–38.88)	9.40^+^ (5.30–15.90)	0.49^+^ (0.29–0.66)	934.40 (842.85–1,048.45)	1.90^+^ (1.20–3.40)
	>75%	16	24.20 (15.80–34.00)	7.20 (4.33–11.60)	0.42 (0.28–0.59)	957.45 (876.60–1,045.43)	2.60 (1.50–4.08)

Multiple rank-sum test:

* Breast milk ratio <50% vs. Breast milk ratio 50%–75% (adj. *P *< 0.05).

# Breast milk ratio <50% vs. Breast milk ratio >75% (adj. *P *<* *0.05).

+ Breast milk ratio 50%–75% vs. Breast milk ratio >75% (adj. *P *<* *0.05).

### Effect of hyperbilirubinemia and phototherapy on bowel sound parameters

There was no difference in the five parameters of bowel sounds between the control group, which consisted of 10 term neonates without hyperbilirubinemia, and the hyperbilirubinemia neonates in the study group (Table 4).

**Table 4:** Comparison of bowel sounds between the hyperbilirubinemia group and the control group.

	Testing age (days), median [IQR]	Total bilirubin (umol/L), median [IQR]	Rate, mean ± SD	Duration, mean ± SD	Amplitude, median [IQR]	Interval, mean ± SD	Frequency, mean ± SD
Control group (*n* = 10)	5.5 [3.5, 6.0]	188.7 [84.6, 273.1]	26.68 (4.56)	9.24 (2.70)	0.44 [0.38, 0.51]	2.81 (0.730)	971.66 (70.70)
Hyperbilirubinemia group (*n* = 68)	6.0 [5.0, 8.0]	298.4 [258.2, 333.5]	27.60 (5.49)	10.12 (2.85)	0.51 [0.41, 0.66]	2.69 (0.714)	956.99 (59.84)
*P*	0.181	0.001	0.639	0.395	0.472	0.649	0.513

After excluding cases without phototherapy or with phototherapy throughout, 50 infants underwent varying durations of phototherapy during the 20 h of bowel sound monitoring. we compared bowel sound parameters between phototherapy or non-phototherapy status in hyperbilirubinemia patients using a paired *t*-test and discovered no significant differences (Table 5).

**Table 5:** Self comparison of bowel sounds between phototherapy or non-phototherapy status during bowel sound monitoring in hyperbilirubinemia patients.

	Photo-Therapy (hours)	Non-photo-Therapy (hours)	Mean of differences	SD	95%CI	*t*	*P*
Rate	10 [7.5, 10.5]	10 [8.0, 11]	−0.88	5.73	−2.51, 0.75	−1.08	0.28
Duration			−0.49	3.18	−1.40, 0.41	−1.09	0.28
Amplitude			−0.03	0.18	−0.08, 0.02	−1.28	0.21
Interval			0.10	0.80	−0.13, 0.32	0.85	0.40
Frequency			−13.43	73.96	−34.45, 7.58	−1.28	0.21

### Safety outcomes

On the skin attached and covered by a sensor patch or surgical dressings, a few infants (*n* = 3) experienced rashes. All rashes vanished within 0.5–2 h of the patch and dressings being removed. Other negative incidents did not happen.

## Discussion/conclusion

The auscultation of bowel sounds has long been regarded as a crucial diagnostic method to assess gastrointestinal function because it is noninvasive, fast, and affordable (16). Conventional auscultation, however, is fundamentally limited by the inability to record detected sounds for review and sharing, as well as the fact that the interpretation of auscultation sounds changes with the skill and experience of the doctor (3, 15). Improving auscultation technology is highly needed in order to provide early diagnosis and accurate monitoring using precision auscultation (3). As a result, we are the first to utilize a wearable acoustic recording and analysis system as a tool for quantitatively evaluating intestinal sound in neonates. According to the results of our investigation, bowel movements can be successfully and securely recorded over time, and the data can subsequently be wirelessly transferred in real time with little noise. Five parameters of bowel sound can be extracted from this data using a CNN visualization technique: rate, duration, amplitude, frequency, and interval.

Neonatal gastrointestinal motility patterns and bowel sound characteristics are different from those of adults since their digestive systems are still developing (17, 18). However, little study has been done on the physiological characteristics and related complications of neonatal bowel sounds. The technique has been used in adult medicine and surgery (11), with an accuracy of 91.8% and the greatest sensitivity of 97.0% (14). However, the viability of utilizing it in neonates was unknown. Given that they can't be compelled to remain still and that they may cry or move more to produce noise and interference, neonates may be more challenging to research than adults. The noise produced by neonatal incubators, phototherapy devices, and monitors also amplifies the sound and interferes with recording equipment (19). Hence, we combined a wireless bowel sound sensor with a specially designed hydrogel patch that can be securely attached to the abdomen for a long period of time and is only 10 grams in weight. The patch is easy to apply and remove without irritating the skin, and it provides excellent skin contact for a high-quality and low-noise signal. The possible impact of the electrical impulses from the heart and breath sounds must be considered in addition to external noise and interference. The acoustic recorder based on CRNN has good sensitivity and specificity in adults and can greatly reduce noise (13). We set a chest control and a blank control while listening to the bowel sounds in order to evaluate the sensitivity and specificity of its application in neonates. The findings demonstrated that the five bowel sound characteristics varied significantly between three record sites. The results imply that the electro-physiological characteristics of neonatal bowel sound parameters are distinct, and that an intelligent acoustic recorder is capable of successfully differentiating and identifying the impact of background noise as well as heart and breath sounds. Therefore, it is now possible to use this technology on neonates, which appears to overcome the limitations of traditional auscultation methods.

Traditional auscultation can only detect one aspect of bowel sounds: the rate, which in healthy adults ranges from 5 to 35 times per minute (20, 21) or every 5 to 10 s (22). The frequency and quality of bowel sounds vary greatly, and they grow more pronounced and frequent after meals (15). According to our research, term infants had more bowel sounds than adults, 25.80 times per minute (IQR: 15.63 to 36.20). This outcome was consistent with the loud, high-frequency bowel sounds detected during auscultation examinations in clinical practice. Additionally, it was also in line with a finding by Richburg (23), who noted that on the fifth day after birth, the cumulative motility of the gut was 41 ± 20 as measured by the total number of bowel movements in four abdominal quadrants within 30 s. This mismatch between neonates and adults may be brought on by the rapid growth and development of neonates, which impose high nutritional requirements, different feeding behaviors (8–12 meals per day, and pure liquid food), and the developing neurological system.

Sakurai et al. (18) used non-digital methods to analyze neonatal bowel sounds after a meal and found that the duration was 12.8 ± 0.31 ms per bowel sound, substantially less than the duration of 8 s/min in our study (converted to duration per bowel sound is 366.81 ms). Ching (24) used an electronic stethoscope to measure the length and found that it was 640 ms (200–1,570) in adults without intestinal obstruction, which is more in accord with our findings. Using computerized auscultation, other researchers (25) discovered that the duration in normal people is approximately 23 ms. They (23) also discovered that the bowel sound interval was 1.931 ± 0.365 s in healthy individuals who had fasted, which was nearly identical to the 2.12 s in neonates in the present study. Both numbers exceed the 0.72 s (0.46–1.27) that Ching (24) found in the obstruction-free control group. The glaring discrepancies in these results could be due to a variety of study factors, such as the techniques and equipment used, the sensitivity and specificity of the sensors, and the timing of bowel sound recording. This might invalidate the comparison. It is crucial to compare people using cutting-edge approaches to the same scenario.

The majority of bowel sounds in neonates are low-frequency sounds with frequencies under 1,000 Hz (18), which included our observations at 944.05 Hz. When Ortigoza (26) employed 300–500 Hz as the dominant frequency to examine the connection between bowel sounds and gastrointestinal maturity in premature infants, he unintentionally made the discovery that the dominant frequency in neonates may be higher than 500 Hz. According to research from adults (24, 25), lower frequencies (200–300 Hz) appear to prevail in bowel sounds. To ascertain whether these variations are brought on by various populations or for other causes, more investigation is needed.

We normalized the observed raw voltage data in terms of amplitude so that all amplitudes were within ±1 after signal processing and 16-bit precision quantization (the range value was within the 15th power of ±2 up and down the zero axis). The amplitude of the typical bowel sound was 0.46. Effective amplitude and frequency can be used to quantitatively define intestinal motility, while the length of intraabdominal sound events can be utilized to qualitatively assess the gastrointestinal tract's evacuation function (27).

In terms of the influencing factors, when comparing the effects of different breast milk intake ratios on bowel sound, we found that the 50%–75% breast milk consumption group had the highest rate, the longest duration, and the largest amplitude of bowel sounds. This runs counter to research showing that a diet high in human milk helps preterm children have better bowel movements (28). This could be a result of different study populations, and premature infants may benefit more from human milk due to their undeveloped gastrointestinal systems. The complexity of regulating gastrointestinal activity and the diverse study approaches may also be to blame for this discrepancy.

Considering the accessibility of the study population, we chose hospitalized term infants with hyperbilirubinemia rather than healthy normal infants, which is the primary limitation of this study. There is no strong evidence for a significant effect of hyperbilirubinemia and phototherapy on neonatal bowel sounds. Previous studies (29) have found that watery greenish stools with increased frequency have been observed in neonates when phototherapy is ongoing. But subsequent studies found that no significant difference was detected in contractile responses of colon segments between control and phototherapy groups (12). Increased stool frequency was considered as a result of the anti-absorptive and elevated gut hormone effects of phototherapy instead of increased gastrointestinal motility (12). Our data analysis also found that there was no significant effect of jaundice and phototherapy on bowel sounds. Naturally, we only included patients with mild to moderate jaundice; further research is required to determine whether severe jaundice has any impact.

Our study also has other limitations. Only the lower right abdomen's bowel noises were being monitored. It is necessary to compare bowel sounds from different abdominal quadrants. Our further research will concentrate on potential influencing factors, whether bowel sound characteristics in neonates are gestationally and postnatally age-specific, and the importance of the technology's use in neonatal clinical settings.

In conclusion, bowel sound monitoring in neonates using a continuous, invasive, and real-time technique looks beneficial when employing a CNN-based intelligent acoustic recorder. Using this technology, we were able to pinpoint the basic acoustic characteristics of bowel sounds in term infants.

## Data Availability

The original contributions presented in the study are included in the article/Supplementary Material, further inquiries can be directed to the corresponding author/s.
